# Effect of ozonated water, mancozeb, and thiophanate-methyl on the phyllosphere microbial diversity of strawberry

**DOI:** 10.3389/fpls.2022.967797

**Published:** 2022-09-15

**Authors:** Ping Sun, Jiaqi Wu, Xianrui Lin, Yi Wang, Jianxi Zhu, Chenfei Chen, Yanqiao Wang, Huijuan Jia, Jiansheng Shen

**Affiliations:** ^1^Jinhua Academy of Agricultural Sciences, Zhejiang Institute of Agricultural Machinery, Jinhua, China; ^2^College of Agriculture and Biotechnology, Zhejiang University, Hangzhou, China

**Keywords:** strawberry, phyllosphere microorganisms, ozonated water, mancozeb, thiophanate-methyl

## Abstract

Phyllosphere microorganisms are closely linked to plant health. This study investigated the effect of ozonated water, mancozeb, and thiophanate-methyl on phyllosphere microorganisms in strawberry plants of the “Hongyan” variety. Sequencing analysis of the phyllosphere bacterial and fungal communities was performed using 16S rRNA gene fragment and ITS1 region high-throughput sequencing after spraying ozonated water, mancozeb, thiophanate-methyl, and clear water. Proteobacteria, Actinobacteria, and Firmicutes were the dominant bacterial phyla in strawberry. The relative abundance of Proteobacteria (82.71%) was higher in the ozonated water treatment group than in the other treatment groups, while the relative abundance of Actinobacteria (9.38%) was lower than in the other treatment groups. The strawberry phyllosphere fungal communities were mainly found in the phyla Basidiomycota and Ascomycota. The relative abundance of Basidiomycota was highest in the ozonated water treatment group (81.13%), followed by the mancozeb treatment group (76.01%), while the CK group only had an abundance of 43.38%. The relative abundance of Ascomycota was lowest in the ozonated water treatment group (17.98%), 23.12% in the mancozeb treatment group, 43.39% in the thiophanate-methyl treatment group, and 55.47% in the CK group. *Pseudomonas*, *Halomonas*, and *Nesterenkonia* were the dominant bacterial genera on strawberry surfaces, while *Moesziomyces*, *Aspergillus*, and *Dirkmeia* were the dominant fungal genera. Ozonated water was able to significantly increase the richness of bacteria and fungi and decrease fungal diversity. However, bacterial diversity was not significantly altered. Ozonated water effectively reduced the relative abundance of harmful fungi, such as *Aspergillus*, and *Penicillium*, and enriched beneficial bacteria, such as *Pseudomonas* and *Actinomycetospora*, more effectively than mancozeb and thiophanate-methyl. The results of the study show that ozonated water has potential as a biocide and may be able to replace traditional agents in the future to reduce environmental pollution.

## Introduction

The living environment of phyllosphere microorganisms is directly exposed to air, and the community structure is directly influenced by factors such as light, humidity, and cultivation measures and indirectly by plant secondary metabolites, resulting in an unstable community structure (Finkel et al., 2012; Vorholt, 2012). Studies have shown that phyllosphere microorganisms can improve resistance to pests and diseases through the production of antimicrobial compounds, microbial–microbial competition, or the activation of plant defenses (Chen et al., 2020). The microbial communities of tomato leaves have been found to protect plants from infection by *Pseudomonas syringae* (Berg and Koskella, 2018). The composition and diversity of the phyllosphere fungal communities of *Eucalyptus grandis* have also been shown to be affected by *Leptocybe invasa* infestation, with significant differences between different levels of infestation (Messal et al., 2022). *Arabidopsis thaliana* phyllosphere-symbiotic *Pseudomonas* strains have even been observed to induce selective inhibition of specific *Arabidopsis* pathogens, leading to plant protection (Shalev et al., 2022). In addition, pathogenic bacteria parasitic on leaves can enter the plant through stomata or wounds when the dynamic balance of the phyllosphere microbial community structure is disturbed, thus inducing disease. Therefore, maintaining the dynamic balance of the phyllosphere microbial community structure and reducing the relative abundance of pathogenic bacteria are of great importance for plant health.

Strawberry (*Fragaria* × *ananassa* Duch.) is a perennial herb of the genus *Fragaria* in the family Rosaceae, native to South America, and is now cultivated around the world. Strawberries are not only unique in flavor but also rich in compounds such as folic acid, ellagic acid, and flavonoids, making them popular among consumers (Hannum, 2004; Tulipani et al., 2009). In 2020, the global output of strawberries reached 8.86 million tons, but strawberry plants are often stressed by biotic or abiotic factors during the cultivation process, which seriously affects yield and quality (Olimi et al., 2022). Traditional chemicals can cause problems such as food safety and environmental pollution, and their use in production has been gradually limited (Sylla et al., 2013). Therefore, the search for new fungicides is imminent.

Ozone is a strong oxidant that is easily soluble in water and has antibacterial and antifungal effects (Inatsu et al., 2011). Ozone decomposes quickly under ambient conditions, thereby having a reduced impact on air and water pollution (Remondino and Valdenassi, 2018). In cultivation, ozone is often used to fumigate the soil to eliminate parasites and pathogenic bacteria, which can effectively reduce the incidence of soil-borne diseases (Nicol et al., 2011). After harvest, ozone can be used to destroy pathogenic bacteria on fruit surfaces and degrade residual pesticides (Wang and Chen, 2020). Ozonated water can inhibit the conidial dispersal of *Phaeoacremonium aleophilum* and reduce the infection rate in grapevine (Pierron et al., 2015). Ozone disinfection of rice seeds effectively inactivates the spores of *Fusarium fujikuroi* (the fungus that causes rice bakanae disease) (Kang et al., 2015). In barley storage, ozone is very effective in inactivating barley-associated fungi, and mycelium is less resistant to ozone than spores (Allen et al., 2003). The use of ozonated water irrigation can significantly increase the shelf life of strawberries, total sugar content, total protein content, and mineral content and reduce the incidence of gray mold, powdery mildew, and other diseases. However, the effects of ozonated water on microorganisms have not been studied in detail (Zhuo et al., 2017; Lu et al., 2018). In this study, strawberry leaves were sprayed with ozonated water, mancozeb, thiophanate-methyl, and clear water, and the effects of different treatments on the structure, composition, and diversity of strawberry phyllosphere microbial communities were investigated using high-throughput sequencing, comparing the differences between ozonated water and traditional agents, which is beneficial to strawberry disease control and provides theoretical support for green production.

## Materials and methods

### Sample source and collection method

The experiment was carried out at the scientific research base of Jinhua National Agricultural Science and Technology Park (119°37′12″ E, 29°01′4.79″ N) on October 22, 2021, and all strawberry plants tested were of the “Hongyan” variety. Strawberry seedlings were preselected for comparable growth rates. Based on the results of the pre-experiment, the ozonated water concentration was chosen to be 3–4 mg/L. Ozonated water above this concentration inhibited strawberry growth, and below this concentration, the effect was not significant. The concentrations of mancozeb and thiophanate-methyl were set according to the product instructions. Four treatments were set up in the test: 3–4 mg/L ozonated water (ozonated water), 70% Mancozeb WP 600 times (mancozeb), 500 g/L Thiophanate-methyl 600 times (thiophanate-methyl), and unaltered water spray was used as the control (CK).

Using a randomized block group design, each treatment was replicated 3 times, with 15 strawberries per replicate, and sprayed once, and strawberry leaves (approximately 50 g) were randomly collected from each group 3 days after application. After the samples were collected, they were placed in sterile sample bags and sent to the laboratory for experimental analysis.

### DNA extraction

Total genomic DNA samples were extracted using the OMEGA Soil DNA Kit (M5635-02) (Omega Bio-Tek, Norcross, GA, United States), following the manufacturer’s instructions, and stored at –20°C prior to further analysis. The quantity and quality of the extracted DNA were measured using a NanoDrop NC2000 spectrophotometer (Thermo Fisher Scientific, Waltham, MA, United States) and 1.0% agarose gel electrophoresis, respectively.

### 16S rRNA gene fragment and ITS1 region amplicon sequencing

Polymerase chain reaction (PCR) amplification of the bacterial 16S rRNA gene fragment in the V5–V7 region was performed using the forward primer 799F (5′-AACMGGATTAGATACCCKG-3′) and the reverse primer 1193R (5′-ACGTCATCCCCACCTTCC-3′). The fungal ITS1 region was amplified using forward primer ITS1F (5′-CTTGGTCATTTAGAGGAAGTAA-3′) and reverse primer ITS2R (5′-GCTGCGTTCTTCATCGATGC-3′). Sample-specific 7-bp barcodes were incorporated into the primers for multiplex sequencing. The PCR components contained 5 μL of reaction buffer (5×), 5 μL of GC buffer (5×), 2 μL of dNTP (2.5 mM), 1 μL of each forward primer (10 μM) and reverse primer (10 μM), 2 μL of DNA template, 0.25 μL of Q5 DNA polymerase, and 8.75 μL of ddH_2_O. Thermal cycling consisted of initial denaturation at 98°C for 2 min, followed by 30 cycles consisting of denaturation at 98°C for 15 s, annealing at 55°C for 30 s, and extension at 72°C for 30 s, with a final extension of 5 min at 72°C. PCR amplicons were purified with Vazyme VAHTSTM DNA Clean Beads (Vazyme, Nanjing, China) and quantified using a Quant-iT PicoGreen dsDNA Assay Kit (Invitrogen, Carlsbad, CA, United States). After the individual quantification step, amplicons were pooled in equal amounts, and paired-end 2 × 250 bp sequencing was performed using the Illumina MiSeq platform with the MiSeq Reagent Kit v3 at Shanghai Personal Biotechnology Co., Ltd. (Shanghai, China).

### Sequence and statistical analysis

Microbiome bioinformatics was performed with QIIME2 2019.4 with slight modifications according to official tutorials^1^ (Bokulich et al., 2018). Briefly, raw sequence data were demultiplexed using the demux plugin, followed by primer cutting with the cutadapt plugin (Martin, 2011). Sequences were then quality filtered, denoised, and merged, and chimeras were removed using the DADA2 plugin (Callahan et al., 2015). Sequence data analyses were performed using the QIIME2 and R packages (v3.2.0). Alpha diversity metrics (Chao1, Shannon, Simpson) and beta diversity metrics (Bray–Curtis dissimilarity) were estimated using the diversity plugin, with samples rarefied to 345 sequences per sample. Taxonomy was assigned to ASVs using the classify-sklearn naïve Bayes taxonomy classifier in the feature-classifier plugin against the SILVA (release 132) and UNITE databases (release 8.0) (Bokulich et al., 2018). A linear discriminant analysis of the effect size (LEfSe) was performed to detect differentially abundant taxa across groups using the default parameters (LSD > 2, *P* < 0.05) (Segata et al., 2011).

## Results and analysis

### Diversity analysis of phyllosphere microbial community in strawberry with different treatments

The alpha diversity indices of bacteria and fungi are shown in Table 1. The Chao1 index showed the highest bacterial abundance after ozonated water treatment (795.41 ± 92.67) and the lowest bacterial abundance after thiophanate-methyl treatment (557.18 ± 120.97), and there was a significant difference between them. The Chao1 index of the ozonated water treatment group was significantly higher than that of the CK group (573.09 ± 221.15). After spraying mancozeb, the Chao1 index was higher than that in the thiophanate-methyl group, but there was no significant difference between them. There was no significant difference in the Shannon and Simpson indices for each treatment group.

**TABLE 1:** Bacterial and fungal alpha diversity indices.

	Bacteria			Fungi		
	Chao1 index	Shannon index	Simpson index	Chao1 index	Shannon index	Simpson index
Ozonated water	795.41 ± 92.67a	5.81 ± 1.24a	0.87 ± 0.12a	57.99 ± 19.1 a	2.10 ± 0.74b	0.45 ± 0.14b
Mancozeb	701.24 ± 85.17abc	6.03 ± 0.53a	0.93 ± 0.03a	44.64 ± 12.17a	3.42 ± 1.17a	0.75 ± 0.24a
Thiophanate-methyl	557.18 ± 120.97bc	5.67 ± 1.13a	0.90 ± 0.12a	44.04 ± 14.56a	2.26 ± 0.97ab	0.56 ± 0.21ab
CK	573.09 ± 221.15c	5.63 ± 1.23a	0.91 ± 0.10a	49.13 ± 11.60a	3.24 ± 0.59ab	0.75 ± 0.10a

The Chao1 indices of strawberry phyllosphere fungi in each treatment group were, in descending order, ozonated water treatment group, CK group, mancozeb treatment group, and thiophanate-methyl treatment group but there was no significant difference between them. The Shannon and Simpson indices were both descending ordered as the mancozeb treatment group, CK group, thiophanate-methyl treatment group, ozone treatment group. The Shannon index was significantly higher in the mancozeb treatment group (3.42 ± 1.17) than in the ozonated water treatment group (2.10 ± 0.74). The Simpson index was significantly higher in both the mancozeb treatment group (0.75 ± 0.24) and CK group (0.75 ± 0.10) than in the ozonated water treatment group (0.45 ± 0.14).

**FIGURE 1:**
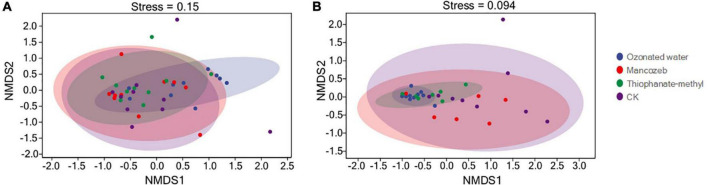
Analysis of microbial beta diversity among different treatment groups. **(A)** Non-metric multidimensional scaling (NMDS) analysis of bacteria; **(B)** NMDS analysis of fungi.

β-diversity analysis was used to compare microbial community differences between treatments (Figure 1). Both bacterial and fungal NMDS analyses showed that the four treatment groups clustered together and could not be distinguished. This indicated that the bacterial and fungal compositions of the four treatment groups were not significantly different. However, the points in the ozonated water treatment group were more concentrated than in the remaining three groups.

### Analysis of phyllosphere bacterial community composition of strawberry plants with different treatments

**FIGURE 2:**
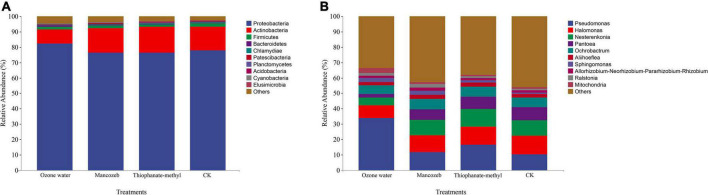
Relative abundance map of phyllosphere bacteria in different treatment groups. **(A)** Relative abundance at the phylum level; **(B)** relative abundance at the genus level.

The sequencing data showed that a total of 16,628,293 high-quality reads were obtained from the four treatments, and a total of 2,791 ASVs were obtained after splicing and quality control. After species annotation, the top 10 phyla and genera of relative abundance were selected for analysis (Figure 2), among which the top 10 phyla of relative abundance were Proteobacteria, Actinobacteria, Firmicutes, Bacteroidetes, Chlamydiae, Patescibacteria, Planctomycetes, Acidobacteria, Cyanobacteria, and Elusimicrobia. The relative abundance of Proteobacteria was the highest in the ozone treatment group (82.17%), while there was little difference among the other treatment groups. The relative abundances of Actinobacteria in the mancozeb treatment group, the thiophanate-methyl treatment group, and the CK group were all above 15%, with little difference, while the relative abundance in the ozonated water treatment group was lower (9.38%). The relative abundances of Firmicutes in each treatment group from large to small were as follows: CK group (2.27%), mancozeb treatment group (1.87%), thiophanate-methyl treatment group (1.61%), and ozonated water treatment group (1.53%).

At the genus level, the relative abundance of *Pseudomonas* in the ozonated water treatment group was significantly higher than that in the other groups. The relative abundance of *Halomonas* was lowest in the ozonated water treatment group (8.33%) and highest in the CK group (12.02%). The relative abundance of *Nesterenkonia* in the ozonated water treatment group (48.82%) was significantly lower than that in the thiophanate-methyl treatment group (11.43%). The relative abundance of *Pantoea* in the ozonated water treatment group (2.27%) was significantly lower than that in the other treatment groups. The relative abundance of *Sphingomonas* in the CK group (0.80%) was significantly lower than that in the ozonated water (2.60%) and mancozeb treatment groups (2.54%). The relative abundance of *Mitochondria* in the ozonated water treatment group (3.05%) was significantly higher than that in the other treatment groups.

### Analysis of phyllosphere fungal community composition of strawberry plants after different treatments

**FIGURE 3:**
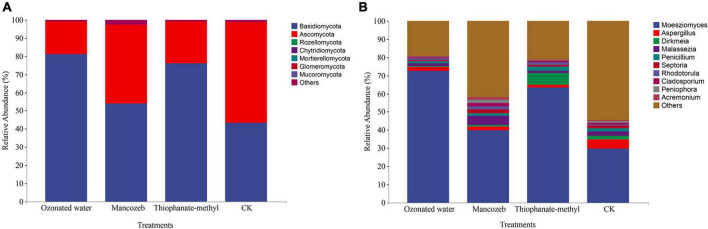
Relative abundance map of phyllosphere fungi in different treatment groups. **(A)** Relative abundance at the phylum level; **(B)** relative abundance at the genus level.

The sequencing data showed that a total of 4,492,346 high-quality reads were obtained from the four treatments, and a total of 392 ASVs were obtained after splicing and quality control. After species annotation, at the phylum level, the strawberry phyllosphere fungal community was mainly comprised of Basidiomycota and Ascomycota, and the relative abundances of the other phyla were all less than 0.01% (Figure 3A). The relative abundance of Basidiomycota in the ozonated water treatment group was the highest, and the CK group was the lowest. The relative abundance of Ascomycota reached 55.47% in the CK group; the relative abundance in the mancozeb treatment group (43.39%) was significantly higher than that in the thiophanate-methyl treatment group (23.13%), and the ozonated water treatment group had the lowest relative abundance (17.89%).

The fungal composition among the treatment groups was further analyzed at the genus level. The results are shown in Figure 3B. *Moesziomyces*, *Aspergillus*, *Dirkmeia*, *Malassezia*, and *Penicillium* were the top five genera in relative abundance in each group. The relative abundance of *Moesziomyces* significantly differed among the groups: from large to small, ozonated water treatment group (72.68%), thiophanate-methyl treatment group (63.52%), mancozeb treatment group (39.73%), and CK group (29.72%). The relative abundance of *Aspergillus* in the CK group (5.02%) was significantly higher than that in the other treatment groups. The relative abundance of *Dirkmeia* was the highest (6.47%) in the thiophanate-methyl treatment group and the lowest (0.46%) in the ozonated water treatment group. The relative abundance of *Malassezia* in the mancozeb treatment group reached 4.89%, which was higher than in the other three treatment groups. The relative abundance of *Penicillium* in the thiophanate-methyl treated group was relatively high (2.48%), while the relative abundance of *Penicillium* in the ozonated water treatment group was low (1.11%).

### Comparison of the relative abundance of strawberry pathogens in different treatment groups

The genera of the pathogenic bacteria of the main strawberry diseases in southern China were selected for analysis. The results are shown in Table 2. *Xanthomonas* had the highest relative abundance in the CK group and the lowest relative abundance in the mancozeb treatment group. The relative abundance of *Pseudocercospora* was zero in the mancozeb group and 0.000027 in the ozonated water treatment group. The relative abundance of *Colletotrichum* was 0.001747, 0.005743, and 0.001838 in the ozonated water, mancozeb, and thiophanate-methyl treatment groups, respectively. The relative abundance of *Pestalotiopsis* was 0 in the ozonated water and thiophanate-methyl treatment groups and 0.003497 in the mancozeb treatment group. The relative abundance of *Botrytis, Podosphaera*, and *Sphaerotheca* was 0 in all treatment groups.

**TABLE 2:** Relative abundance of strawberry pathogens.

	Genus	Ozonated water	Mancozeb	Thiophanate-methyl	CK
Bacteria	*Xanthomonas*	0.000081	0.000007	0.000448	0.000560
Fungi	*Pseudocercospora*	0.000027	0.000000	0.000257	0.000981
	*Colletotrichum*	0.001747	0.005743	0.001838	0.000895
	*Pestalotiopsis*	0.000000	0.003497	0.000000	0.000026
	*Botrytis*	0.000000	0.000000	0.000000	0.000000
	*Podosphaera*	0.000000	0.000000	0.000000	0.000000
	*Sphaerotheca*	0.000000	0.000000	0.000000	0.000000

### Differential analysis of phyllosphere microorganisms on strawberry plants after different treatments

**FIGURE 4:**
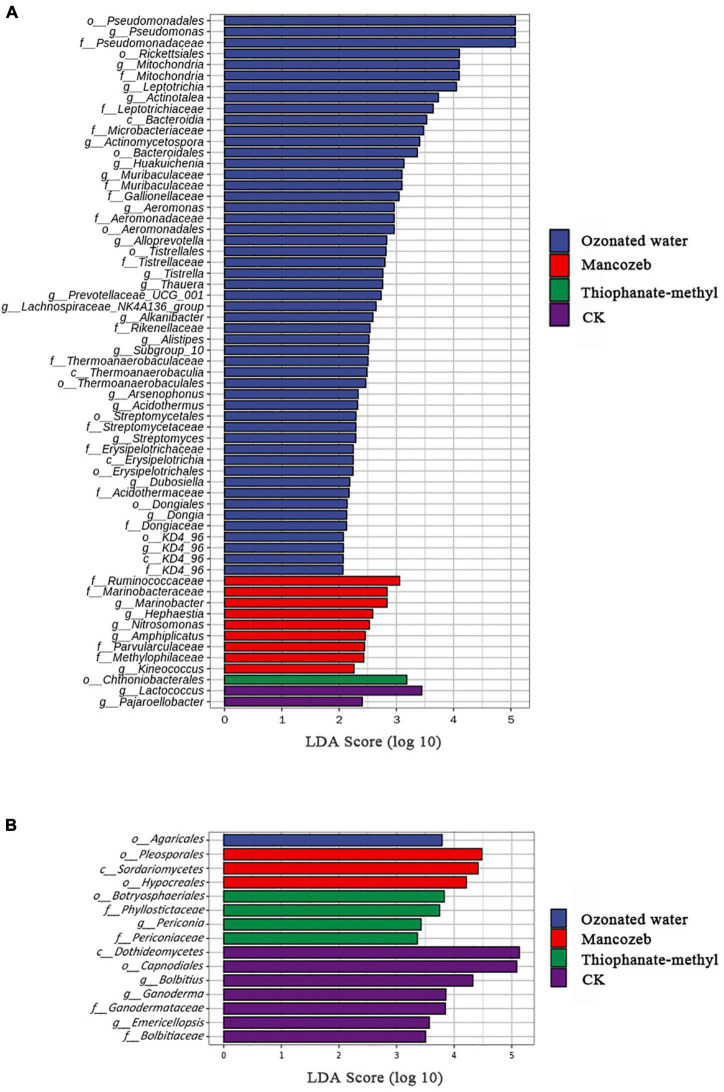
Linear discriminant analysis effect size (LEfSe) analysis of phyllosphere microorganisms of different treatments: **(A)** bacteria and **(B)** fungi.

LEfSe analysis was used to analyze the enriched characteristic groups in different treatments, with the linear discriminant analysis threshold set to 2. The bacterial results are shown in Figure 4A. The ozonated water treatment group had the most biomarkers, including Bacteroidia, Thermoanaerobaculia, Erysipelotrichia, and KD4_96; Pseudomonadales, Rickettsiales, Bacteroidales, and seven other orders; Pseudomonadaceae, Mitochondria, Leptotrichiaceae, and 12 other families; and *Pseudomonas*, *Mitochondria*, *Leptotrichia*, and 19 other genera. The mancozeb treatment group had nine biomarkers, including Ruminococcaceae Marinobacteraceae, Parvularculaceae, Methylophilaceae, and five genera. *Chthoniobacterales* is a biomarker for the thiophanate-methyl treatment group. *Lactococcus and Pajaroellobacter* were biomarkers for the CK group.

When the relative abundance of fungal communities in the different treatment groups was compared, the threshold was set to 2. The results are shown in Figure 4B. A total of 15 biomarkers were present in the different treatment groups. The highest number of biomarkers was found in the CK group, including Dothideomycetes, Capnodiales, Ganodermataceae, Bolbitiaceae, Bolbitius, Ganoderma, and Emericellopsis. Botryosphaeriales, Phyllostictaceae, Periconiaceae, and Periconia were included in the thiophanate-methyl treatment group. The mancozeb treatment group included Sordariomycetes, Hypocreales, and Pleosporales. The ozonated water treatment group had the fewest biomarkers, only Agaricales.

## Discussion

### Effects of different treatments on the diversity of the phyllosphere microbial community of strawberry plants

The living environment of phyllosphere microorganisms changes rapidly, and factors such as agricultural cultivation measures, plant genotypes, and developmental stages affect the abundance and diversity of phyllosphere microorganisms (Whipps et al., 2008; Bringel and Couee, 2015; Lajoie et al., 2020). Bacteria and fungi are two types of phyllosphere microorganisms that researchers have focused on. Microbial–biological and microbe–host-plant interactions affect the fitness of plants in nature, the yield of crops, and the safety of horticultural products (Whipps et al., 2008). Bacteria and fungi are the main phyllosphere microorganisms of strawberry plants, and most diseases of strawberry plants are directly related to fungi, with the yield and quality of strawberries greatly reduced when the disease is severe (Amil-Ruiz et al., 2011; Holmes et al., 2020). *Fusarium oxysporum* can cause strawberry wilt, which causes the plant to collapse and die (Koike and Gordon, 2015). The main pathogens of strawberry anthracnose are the following three species: *Colletotrichum fragariae*, *C. gloeosporioides*, and *C. acutatum*, which can lead to plant death in severe cases (Brooks, 1931; Arroyo et al., 2007; Widiastuti et al., 2013). Powdery mildew is one of the main facility diseases of strawberry, mainly caused by *Podosphaera aphanis* (Feng et al., 2020). After spraying leaves with ozonated water, the richness of bacteria and fungi in the strawberry phyllosphere significantly increased, and the diversity of fungi was reduced, but the effect on bacterial diversity was not significant. Spraying mancozeb increased the bacterial richness and fungal diversity of strawberry leaves and reduced fungal richness and bacterial diversity, but the effect was not significant. The application of thiophanate-methyl had no significant effect on the bacterial and fungal richness and diversity of the strawberry phyllosphere. As a broad-spectrum green fungicide, ozonated water has a good bactericidal effect, but it also has a negative impact on beneficial bacteria. After spraying ozonated water, the richness of bacteria and fungi in the strawberry phyllosphere increased, which may be because, after ozonated water depopulated the phyllosphere, it was repopulated from the surrounding soil and air. Because the collected samples were in the same environment, the microorganisms recolonizing the leaves were similar, and the points causing ozonated water in the NMDS analysis were more concentrated compared to the other treatment groups. As a protective fungicide, mancozeb can form a protective layer on the surface of leaves and fruits, preventing pathogen invasion and reducing the activity of various enzymes required for the normal physiological metabolism of pathogens (Jabeen et al., 2011). Thiophanate-methyl is a systemic broad-spectrum fungicide that can kill microorganisms on the leaf surface (Papadopoulou-Mourkidou, 1991). This prevents the immediate repopulation of the phyllosphere from air and soil microbes, causing a decrease in the richness and diversity of phyllosphere microorganisms.

### Effects of different treatments on the composition of strawberry phyllosphere microbial community

The present study found that *Pseudomonas*, *Halomonas*, *Nesterenkonia*, *Pantoea*, and *Ochrobactrum* were the dominant bacterial genera on strawberry leaves. *Moesziomyces*, *Aspergillus*, and *Dirkmeia* were found to be the dominant fungal genera. This is inconsistent with Olimi et al. (2022), but it may be caused by factors such as cultivation conditions and strawberry variety. At the fungal phylum level, ozonated water treatment decreased the relative abundance of Ascomycota and increased the relative abundance of Basidiomycota. The most common diseases of strawberry plants are fungal diseases (such as anthracnose, powdery mildew, and root rot), and the remaining pathogenic fungi belong to *Ascomycota* (Zhang and Zhong, 2018; Weber and Hahn, 2019; Yang et al., 2019). Therefore, the application of ozonated water sprays can effectively reduce pathogenic fungi populations in strawberry leaves, thereby reducing the incidence of plant diseases. Mancozeb and thiophanate-methyl are both fungicides and have no disinfecting effect on bacteria; thus, compared with water, the difference in bacterial composition is not obvious. Since mancozeb is a protective fungicide, the relative abundance of Ascomycota on the leaf surface is not clearly different from that of the CK group, while thiophanate-methyl is a broad-spectrum fungicide that can directly reduce fungal populations.

Some bacteria of the genus *Pseudomonas*, such as *P*. *fluorescens*, can symbiotically combat plant diseases (Sun et al., 2008; Bai et al., 2014). They can be used as important potassium-solubilizing and scale-solving bacteria in soil to improve soil nutrient utilization (Bai et al., 2014). In the post-harvest preservation of strawberries, apples, and grapes, these bacteria can also effectively reduce the incidence of gray mold and acid rot (Sun et al., 2008; Ilhan and Karabulut, 2013). *Actinomycetospora* has antibacterial activity and has an inhibitory effect on fungi (Phongsopitanun et al., 2020). After applying ozonated water, *Pseudomonas* and *Actinomyces* were significantly enriched in the strawberry phyllosphere, indicating that spraying ozonated water enriched beneficial bacteria and increased the resistance of strawberry plants to several agricultural pathogens.

*Aspergillus* and *Penicillium* are common fungi in the phyllosphere, and fungi from these two genera are often associated with diseases (Wang et al., 2008; Gupta and Rathore, 2013). *Aspergillus* causes mildew in tobacco and is the main fungus responsible for medicinal material decomposition during storage (Guo and Pang, 2018; Chen et al., 2019). *Penicillium* infects the surface of strawberries and produces mycotoxins, causing food-safety hazards (Chao et al., 2017). After applying ozonated water, the relative abundance of *Aspergillus* and *Penicillium* decreased, indicating that spraying ozonated water can kill harmful fungi in strawberry leaves and can effectively prevent pathogens from being transferred from leaves to fruit, thereby extending the shelf life of strawberries. The number of enriched fungi in the CK group was significantly higher than those in the other groups, indicating that ozonated water, mancozeb, and thiophanate-methyl could effectively kill strawberry phyllosphere fungi, with ozonated water showing the most promise.

### Effects of different treatments on strawberry pathogens

*Xanthomonas fragariae* is the causal agent of strawberry bacterial angular leaf spot disease, which produces irregular spots on the leaves after infection and causes blackening and wilting of the eyes of the main stem shoots in severe cases (Kim et al., 2016). *Pseudomonas* and *Rhizobium* were found to inhibit the growth of *X. fragariae* (Henry et al., 2016). The highest relative abundance of *Pseudomonas* was found in the ozonated water treatment group, and the relative abundance of *Xanthomonas* decreased after the application of ozonated water, indicating that ozonated water has the potential to control strawberry bacterial angular leaf spot disease. *Pseudocercospora fragarina* is the causal agent of gray spot disease in strawberry, which occurs mainly in the spring. The relative abundance of *Pseudocercospora* decreased after the application of ozonated water and the chemical mancozeb, which are used in production for the control of gray spots and may explain the low relative abundance of *Pseudocercospora*. In this study, the relative abundance of *Colletotrichum* was found to be higher in all four treatment groups compared to the other strawberry pathogens. The experimental area was located in Jinhua, Zhejiang Province, China, with high precipitation in October and average daytime temperatures of around 24°C, a period of high strawberry anthracnose incidence. Among the three groups of fungicide treatments, the ozonated water treatment group had the lowest relative abundance of *Colletotrichum*, indicating the potential of ozonated water to prevent anthracnose. In contrast, the relative abundance of the control *Colletotrichum* was 0.000895, which may be caused by the decrease in the richness of some other microbes inhibiting *Colletotrichum*. Although the relative abundance of *Colletotrichum* was higher in the ozonated water treatment group and the pharmaceutical treatment group than in the CK group, overall, the relative abundance of *Colletotrichum* was not high in either group, and the strawberry’s own defense mechanisms resisted the invasion of these pathogens, and anthracnose had not yet occurred. The pathogenic fungi of strawberry root rot are more complex, and *Pestalotiopsis* and *Colletotrichum* can cause strawberry root rot (Salinas et al., 2020; Baggio et al., 2021). *Botrytis cinerea* and *P. aphanis* are the causal agents of gray mold and powdery mildew, respectively, in strawberry (Kamaruzzaman et al., 2018; Feng et al., 2020). The study reported that *Sphaerotheca macularis* can also cause the occurrence of powdery mildew in strawberries (Mahmud et al., 2020). These two diseases occur primarily in the spring in Zhejiang Province. In this study, the sampling time was October, when a high incidence of gray mold and powdery mildew was not reached, so pathogenic fungi were not detected.

## Conclusion and prospects

As a green fungicide, ozonated water can effectively reduce the incidence of crop diseases. Our research on the phyllosphere microbial community of strawberry plants under different treatments showed that spraying ozonated water, mancozeb, and thiophanate-methyl did not have a great influence on the diversity of the strawberry phyllosphere microbial community. Ozonated water can effectively reduce the relative abundance of pathogenic fungi (*Aspergillus* and *Penicillium*) in strawberry leaves and foster the growth of beneficial microorganisms, including *Pseudomonas* and *Actinomyces*. Compared with traditional pesticides, ozonated water has a more obvious bactericidal effect and does not pollute the environment. In the future, diseased strawberries can be used to explore the effect of ozonated water on phyllosphere microorganisms, and *in vitro* experiments can be conducted to further explore the effect of ozonated water on strawberry phyllosphere fungi and bacteria.

## Data availability statement

The data presented in this study are deposited in the Genome Sequence Archive, (GSA) repository, accession number: CRA007542.

## Author contributions

PS, XL, JS, and HJ designed the experiments. JW, YiW, CC, JZ, and YqW performed the experiments. PS, JW, and XL analyzed the data and compiled the figures. PS, JW, and JS wrote the manuscript. YqW, HJ, and JS edited the final manuscript. All authors contributed to the article and approved the submitted version.

## Footnotes

1
https://docs.qiime2.org/2019.4/tutorials/
